# Diminishing returns, increasing risks: Impact of antibiotic duration of therapy on respiratory bacterial isolates in hospitalized patients during the coronavirus disease 2019 (COVID-19) pandemic

**DOI:** 10.1017/ash.2021.173

**Published:** 2021-07-23

**Authors:** Catherine Li, Ryan W. Chapin, Nicholas J. Mercuro, Christina F. Yen, Howard S. Gold, Matthew S. L. Lee, Christopher McCoy

**Affiliations:** 1 Department of Pharmacy, Beth Israel Deaconess Medical Center, Boston, Massachusetts; 2 Division of Infectious Diseases, Beth Israel Deaconess Medical Center, Harvard Medical School, Boston, Massachusetts; 3 Silverman Institute for Health Care Quality and Safety, Beth Israel Deaconess Medical Center, Boston, Massachusetts

## Abstract

In 829 hospital encounters for patients with COVID-19, 73.2% included orders for antibiotics; however, only 1.8% had respiratory cultures during the first 3 hospital days isolating bacteria. Case–control analysis of 30 patients and 96 controls found that each antibiotic day increased the risk of isolating multidrug-resistant gram-negative bacteria (MDR-GNB) in respiratory cultures by 6.5%.

Antibiotic prescribing in patients with COVID-19 remains an issue of great importance to antimicrobial stewardship. At present, the World Health Organization recommends limiting antibiotics to patients with severe disease, emphasizing the need for clinical judgment; whereas the Surviving Sepsis Campaign grades the recommendation for empiric antibiotics in mechanically ventilated patients as weak and based on low-quality evidence.^1,2^ Antimicrobial overuse during the COVID-19 pandemic, in combination with lapses in infection prevention measures, has likely contributed to outbreaks of antimicrobial-resistant organisms in the United States and other countries.^3–6^ In this study, we compared rates of antibiotic utilization and bacterial isolation in hospitalized patients with COVID-19, and we characterized the impact of antibiotic exposure on the isolation of multidrug-resistant gram-negative bacteria (MDR-GNB).

## Methods

This retrospective observational study was conducted at a tertiary-care, academic, medical center in Boston, Massachusetts. Patient hospital encounters between March 1 and May 31, 2020 with a positive severe acute respiratory syndrome coronavirus 2 (SARS-CoV-2) nasopharyngeal polymerase chain reaction (PCR) test result or an *International Classification of Disease, Tenth Revision* (ICD-10) code for COVID-19 were included. In-house SARS-CoV-2 PCR testing was implemented using the Aldatu PANDAA qDx platform (Aldatu Biosciences, Watertown, MA) and Abbott RealTime PCR (Abbott Diagnostics, Lake Forest, IL) on March 19 and April 2, 2020, respectively. Patients aged <18 years were excluded. This study was approved by the institutional review board.

Patient characteristics including sex, race, underlying comorbidities, and previous hospitalization within 90 days were collected from an institutional data repository. Data regarding hospital length of stay, *Clostridioides difficile* infection, and inpatient mortality were also collected. Days of antibiotic therapy per 1,000 patient days (pDOT/1,000) was calculated for the following agents with activity against respiratory pathogens: azithromycin, ceftriaxone, cefepime, ceftazidime, piperacillin/tazobactam, meropenem, vancomycin, linezolid, and levofloxacin. Days of therapy were compared to historic institutional usage from March to May in 2018 and 2019.

A positive respiratory culture was defined as growth of at least 1 bacterial species, excluding commensal flora. Bacteria were considered community or hospital-acquired if culture collection occurred within the first 3 calendar days of hospitalization or afterward, respectively. Patients with community-acquired bacteria on cultures were reviewed for transfer from an outside institution to confirm the time from initial admission.

A case–control analysis was conducted to assess the impact of inpatient antibiotic exposure on MDR-GNB isolation during this period. Cases and controls were selected from hospitalized patients with respiratory culture results from March 1 to May 31, 2020. Patients with and without a COVID-19 diagnosis were included to increase generalizability of results and to control for possible confounding from unit-based cohorting during the pandemic. Cases had respiratory cultures isolating MDR-GNB (defined as bacteria resistant to at least one agent in 3 or more antibiotic classes). Controls had commensal flora, yeast, or bacteria that were not multidrug resistant or had no growth in respiratory cultures. A post hoc sensitivity analysis including only patients with COVID-19 was performed. Days of antibiotic therapy with gram-negative activity (amikacin, ampicillin, cefepime, ceftazidime, ceftriaxone, ciprofloxacin, gentamicin, levofloxacin, meropenem, piperacillin/tazobactam, and tobramycin) were collected using the electronic medication administration record. Data regarding select comorbidities, previous hospitalization with receipt of intravenous antibiotics within 90 days, previous MDR-GNB within 360 days, and intensive care unit admission were also collected.

The χ^2^ and Fisher exact test were used for categorical variables, and the Mann-Whitney *U* test was used for continuous variables. Covariates associated with MDR-GNB were identified in univariate analysis and previously published literature and were assessed using logistic regression. Descriptive statistics were applied for all other outcomes. Analyses were conducted using SPSS Statistics version 27.0 software (IBM, Armonk, NY).

## Results

Of 7,969 hospital encounters during the study period, 829 were included, comprising 711 unique patients. Confirmed nasopharyngeal SARS-CoV-2 PCR results were documented in 819 encounters (98.8%), and the remainder had a COVID-19 ICD-10 code. Patient demographics and outcomes are displayed in Supplementary Table 1 (online). Antibiotics were initiated in 607 encounters (73.2%), and utilization in patients with COVID-19 was 831.9 compared to 368.3 pDOT/1,000 across all admitted inpatients. Utilization of azithromycin, ceftriaxone, cefepime, ceftazidime, meropenem, vancomycin, and linezolid increased by at least 2-fold in patients with COVID-19 compared to historical use in 2018 and 2019 (Supplementary Table 2 online). In total, 567 respiratory cultures were collected from 196 encounters (23.6%). Community-acquired bacteria were isolated in 15 patient encounters (1.8%). From the 601 encounters with a hospital stay of at least 4 days, hospital-acquired bacteria were isolated in 58 encounters (9.7%), with a median time to culture positivity of 5 days (interquartile range [IQR], 3–9) after admission.

The case–control analysis included 30 patients isolating MDR-GNB in respiratory cultures and 96 control patients (Table 1). Prior to culture collection, median antibiotic duration was longer in patients isolating MDR-GNB (5.5 days; IQR 1.5–11) compared to the control group (3 days; IQR, 0–7). Cases were also more likely to have prior MDR-GNB isolated within the previous year. After controlling for structural lung disease and recent antibiotic exposure in previous admissions, each day of antibiotic exposure prior to culture collection increased the risk of MDR-GNB isolation from respiratory culture by 6.5% (OR, 1.065; 95% CI, 1.004–0.130) (Table 2). These findings were confirmed in a sensitivity analysis for COVID-19 patients (n = 73), with prolonged prior antibiotic exposures in the MDR-GNR group (10.5 days, [IQR, 5–15.3] vs 3 days [IQR, 1–6]; *P* < .001). After culture collection, median antibiotic duration was similar between groups.

**Table 1.** Characteristics of Patients With and Without Multidrug-Resistant Gram-Negative Bacteria (MDR-GNB) in Respiratory Cultures During March 1–May 31, 2020

Characteristic	MDR-GNB (n = 30)	Control (n = 96)
COVID-19 infection, no. (%)	14 (46.7)	58 (60.4)
Sex, male, no. (%)	21 (70.0)	57 (59.4)
Age, y (SD)	63.5 (13.8)	64.3 (15.0)
**Comorbid conditions, no. (%)**		
Diabetes mellitus	14 (46.7)	34 (35.4)
Obesity	6 (20.0)	22 (22.9)
Congestive heart failure	9 (30.0)	30 (31.3)
Chronic obstructive pulmonary disorder	6 (20.0)	8 (8.3)
**Respiratory culture microbiology, no. (%)**		
MDR-GNB		
*Pseudomonas* spp	10 (33.3)	…
*Escherichia coli*	6 (20.0)	…
*Enterobacter* spp	6 (20.0)	…
*Klebsiella pneumoniae*	3 (10.0)	…
*Achromobacter* spp	2 (6.7)	…
*Acinetobacter baumanii*	2 (6.7)	…
*Chryseobacterium indologenes*	1 (3.3)	…
Non–MDR-GNB		
Enterobacterales	…	35 (36.5)
*Pseudomonas* spp	…	20 (20.8)
*Stenotrophomonas maltophilia*	…	5 (5.2)
*Burkholderia cepacia*	…	1 (1.0)
*Haemophilus influenzae*	…	1 (1.0)
*Staphylococcus aureus*	…	5 (5.2)
Commensal respiratory flora	…	23 (24.0)
Yeast	…	11 (11.5)
**Respiratory culture source, no. (%)**		
Endotracheal sputum	22 (73.3)	70 (72.9)
Bronchoalveolar lavage	4 (13.3)	15 (15.6)
Expectorated sputum	4 (13.3)	11 (11.5)
Hospital length of stay, median d (IQR)	22.5 (14–35.25)	21 (14–35.25)
Intensive care unit admission, no. (%)	30 (100.0)	87 (90.6)
**Days of antibiotics with gram negative activity, median (IQR)**		
Prior to culture collection	5.5 (1.5–11)	3 (0–7)
After culture collection	9 (6.25–14.75)	8 (4–16)
Total	16 (11.25–25)	12 (7–20)
**Location of antibiotic initiation in patients with COVID-19, no. (%)**		
Intensive care unit	8 (57.1)	22 (37.9)
Emergency department	3 (21.4)	17 (29.3)
Medical/surgical unit	1 (7.1)	6 (10.3)
*Clostridioides difficile* polymerase chain reaction–positive	1 (3.3)	3 (3.1)
Inpatient mortality or discharge to hospice	11 (36.7)	29 (30.2)

Note. IQR, interquartile range.

**Table 2.** Exposures Associated With Isolation of Multidrug-Resistant Gram-Negative Bacteria (MDR-GNB) in Respiratory Cultures During March 1–May 31, 2020

Variable	MDR-GNB (n = 30)	Control (n = 96)	*P* Value	Adjusted OR (95% CI)
Chronic obstructive pulmonary disease, no. (%)	6 (20.0)	8 (8.3)	.097	2.538 (0.752–8.570)
Hospital admission in last 90 d, no. (%)	9 (30.0)	17 (17.7)	.146	…
Intravenous antibiotics in 90 d preceding admission, no. (%)	8 (26.7)	12 (12.5)	.064	2.071 (0.711–6.028)
Previous MDR-GNB within 360 d, no. (%)	5 (16.7)	1 (1.0)	.003	…
Antibiotic duration prior to respiratory culture, d (IQR)	5.5 (1–12)	3 (0–7)	.017	1.065 (1.004–1.130)
Total antibiotic duration during hospitalization, d (IQR)	16 (10.5–25)	12 (7–20)	.042	…
Intensive care unit length of stay, median d (IQR)	14.5 (4.25–26.0)	17 (8–25.25)	.801	…

Note. OR, odds ratio; CI, confidence interval; IQR, interquartile range.

## Discussion

Our findings are consistent with other publications of infrequent bacterial coinfection in patients with COVID-19 presenting from the community. A meta-analysis of 24 studies with a total of 3,338 patients reported that 3.5% (95% CI, 0.4–6.7) had bacterial coinfection at hospital presentation. Nevertheless, 71.8% (95% CI, 56.1%–87.7%) of patients received antibiotics.^7^ In our case–control analysis, we identified a relationship between antibiotic duration prior to isolation of multidrug-resistant bacteria in patients with COVID-19, further characterizing potential harms of antimicrobial initiation during the pandemic. Notably, antibiotic exposure was similar between groups after culture collection, highlighting additional stewardship opportunities for antimicrobial de-escalation. Our analysis included patients with and without COVID-19, suggesting that an impact of the pandemic on the healthcare system extended beyond patients diagnosed with COVID-19.

Our single-center analysis has several limitations. Despite the high frequency of antibiotic prescribing, only 23.6% of patient encounters had adequate respiratory samples for culture. This low rate may have been affected by personal protective equipment shortages and concern for infection transmission by aerosol-generating procedures including bronchoscopy.^8^ Additionally, antibiotic indications were presumed to be used for empirical therapy of pneumonia and/or sepsis. Positive respiratory cultures were not correlated with clinical suspicion for coinfection and could represent bacterial colonization, particularly in mechanically ventilated patients. Furthermore, due to the retrospective study design and low overall prevalence of MDR-GNB, potential unmeasured confounders and covariates with low event rates (eg, prior MDR-GNB) limited our analysis.

In conclusion, antibiotics with activity against respiratory pathogens were prescribed during 73.2% of patient hospital encounters for COVID-19, despite only 1.8% having respiratory cultures isolating bacteria within the first 3 hospital days. Furthermore, each day of inpatient antibiotic exposure increased the risk of MDR-GNB isolation in respiratory culture by 6.5%. We have incorporated these findings to support institutional treatment guidelines and to leverage daily antimicrobial stewardship education and interventions in patients with COVID-19. In addition to standard infection control practices, reflexive antibiotic prescribing should be deterred through stewardship efforts and should remain a priority during the COVID-19 pandemic to prevent the accelerated selection of multidrug-resistant organisms.

## Supplementary material

For supplementary material accompanying this paper visit https://doi.org/10.1017/ash.2021.173. click here to view supplementary material

## References

[1] Clinical management of COVID-19 interim guidance. World Health Organization website. https://www.who.int/publications-detail/clinical-management-of-severe-acuterespiratory-infection-when-novel-coronavirus-(ncov)-infection-is-suspected. Published 2020. Accessed June 28, 2021.

[2] Alhazzani W , Møller MH , Arabi YM , et al. Surviving sepsis campaign: guidelines on the management of critically ill adults with coronavirus disease 2019 (COVID-19). Crit Care Med 2020;48:e440e69. 10.1097/CCM.0000000000004363 PMC7176264 32224769

[3] Nori P , Cowman K , Chen V , et al. Bacterial and fungal coinfections in COVID-19 patients hospitalized during the New York City pandemic surge. Infect Control Hosp Epidemiol 2021;42:84–88. 32703320 10.1017/ice.2020.368 PMC7417979

[4] Bork JT , Leekha S , Claeys K , et al. Change in hospital antibiotic use and acquisition of multidrug-resistant gram-negative organisms after the onset of coronavirus disease 2019. *Infect Control Hosp Epidemiol* 2020. doi: 10.1017/ice.2020.1360. PMC7783138 33298211

[5] Tiri B , Sensi E , Marsiliani V , et al. Antimicrobial stewardship program, COVID-19, and infection control: spread of carbapenem-resistant *Klebsiella pneumoniae* colonization in ICU COVID-19 patients. What did not work? J Clin Med 2020;9:2744. 10.3390/jcm9092744 PMC7563368 32854334

[6] Guisado-Gil AB , Infante-Domínguez C , Peñalva G , et al. Impact of the COVID-19 pandemic on antimicrobial consumption and hospital-acquired candidemia and multidrug-resistant bloodstream infections. Antibiotics 2020;9:816. 10.3390/antibiotics9110816 PMC7698104 33212785

[7] Langford BJ , So M , Raybardhan S , et al. Bacterial coinfection and secondary infection in patients with COVID-19: a living rapid review and meta-analysis. Clin Microbiol Infect 2020;26:1622–1629. 32711058 10.1016/j.cmi.2020.07.016 PMC7832079

[8] Tran K , Cimon K , Severn M , Pessoa-Silva CL , Conly J. Aerosol-generating procedures and risk of transmission of acute respiratory infections to healthcare workers: a systematic review. PLoS One 2012;7:e35979. 22563403 10.1371/journal.pone.0035797 PMC3338532

