# Supplementary material for: Diminishing returns, increasing risks: Impact of antibiotic duration of therapy on respiratory bacterial isolates in hospitalized patients during the coronavirus disease 2019 (COVID-19) pandemic
Source: Antimicrob Steward Healthc Epidemiol. 2021 Jul 23;1(1):e13. doi: 10.1017/ash.2021.173 (PMC9495623; doi:10.1017/ash.2021.173)
Supplement: Supplementary file 1 [file S2732494X2100173Xsup001.docx]

Supplemental Table 1: Characteristics for patients admitted with COVID-19 during March 1 to May 31, 2020

|  | n=829 |
| --- | --- |
| Sex, male n (%) | 410 (49.5) |
| Age, years (SD) | 64.9 (17.9) |
| Race       White       Black       Hispanic       Asian/Pacific Islander       Other       Unknown | 314 (37.9)  208 (25.1)  151 (18.2)  41 (4.9)  38 (4.6)  77 (9.3) |
| Comorbid conditions, n (%)       Diabetes mellitus       Hypertension       Obesity       Congestive heart failure       Chronic obstructive pulmonary disorder       End stage renal disease       Human immunodeficiency virus | 341 (41.1)  330 (39.8)  161 (19.4)  159 (19.2)  84 (10.1)  54 (6.5)  2 (0.2) |
| Hospital admission in last 90 days, n (%) | 112 (13.5) |
| **Events after admission** |  |
| Length of stay, days median (IQR) | 6 (2-13) |
| Endotracheal intubation, n (%) | 133 (16.0) |
| Prescribed any antibiotic listed below, n (%)       Ceftriaxone       Vancomycin or linezolid       Anti-pseudomonal beta-lactam       Azithromycin       Levofloxacin | 607 (73.2)  415 (50.0)  273 (32.9)  262 (31.6)  262 (31.6)  40 (4.8) |
| Respiratory culture obtained, n (%)       Endotracheal aspirate       Expectorated sputum       Bronchoalveolar lavage | 196 (23.6)  176 (21.2)  34 (4.1)  13 (1.6) |
| Positive respiratory culture, n (%)       Community-acquired bacteria       Hospital-acquired bacteria* | 73 (8.8)  15 (1.8)  58 (9.7) |
| Microbiology from 567 respiratory cultures, n (%)       Enterobacterales  *Pseudomonas aeruginosa*  *Staphylococcus aureus*  *Streptococcus* species  *Haemophilus influenzae*  *Moraxella catarrhalis*       Commensal respiratory flora       Yeast       No growth | 76 (13.4)  43 (7.6)  75 (13.2)  4 (0.7)  3 (5.3)  1 (0.2)  176 (31.0)  137 (16.5)  46 (8.1) |
| *C. difficile* polymerase chain reaction positive | 29 (3.5) |
| Inpatient mortality or discharge to hospice | 171 (20.6) |

* Out of 601 encounters with a hospital length of stay of at least 4 days

Supplemental Table 2: Antibiotic utilization per 1000 patient-days of therapy for patients with COVID-19 compared to total hospital census from March 1 to May 31 2018, 2019, and 2020

|  | 2020 | | 2019 | 2018 |
| --- | --- | --- | --- | --- |
| Total hospital days | -- | 54394 | 63507 | 62641 |
|  | COVID-19 DOT/1000 | Average DOT/1000 patient days | | |
| Total of all below agents | 831.9 | 368.3 | 281.7 | 274.7 |
| Vancomycin | 190.8 | 74.9 | 50.9 | 57.5 |
| Ceftriaxone | 184.1 | 102.7 | 72.8 | 67.5 |
| Cefepime | 170.2 | 79.0 | 62.7 | 58.0 |
| Azithromycin | 97.5 | 18.4 | 13.8 | 15.0 |
| Ceftazidime | 67.5 | 21.5 | 14.2 | 15.9 |
| Meropenem | 64.4 | 28.7 | 22.4 | 17.7 |
| Levofloxacin | 23.1 | 7.9 | 9.6 | 8.4 |
| Piperacillin/tazobactam | 22.9 | 30.3 | 32.2 | 31.5 |
| Linezolid | 11.4 | 4.8 | 3.1 | 3.1 |
